# Sophorolipid inhibits histamine-induced itch by decreasing PLC/IP3R signaling pathway activation and modulating TRPV1 activity

**DOI:** 10.1038/s41598-023-35158-9

**Published:** 2023-05-17

**Authors:** Rui-Qi Xu, Ling Ma, Timson Chen, Wei-Xiong Zhang, Kuan Chang, Jing Wang

**Affiliations:** 1 Key Laboratory of Synthetic and Biological Colloids, Ministry of Education, School of Chemical and Material Engineering, Jiangnan University, Wuxi, 214122 China; 2 Adolph Innovation Laboratory, Guangzhou Degu Personal Care Products Co., Ltd., Guangzhou, 510000 China

**Keywords:** Biochemistry, Cell biology, Diseases, Health care

## Abstract

Biosurfactants are attracting much interest due to their potential application as therapeutic agents in the medical and cosmetic field. Previous studies have demonstrated that biosurfactant such as sophorolipid (SL) exhibits immunomodulatory effects. In this article, we found the potential of sophorolipid for inhibiting histamine-induced itch and preliminarily explored its molecular basis. First, behavioral tests indicated that SL can remit the histamine-induced scratching behaviors of mice. Second, SL can suppress the the calcium influx induced by histamine, HTMT and VUF8430 in HaCaT cells. RT-PCR analysis showed that the histamine-induced upregulation of mRNA levels of phospholipase Cγ1, 1,4,5-trisphosphate receptor (IP3R), and protein kinase Cα can be inhibted by SL, suggesting that SL may impede the PLC/IP3R signaling pathway activated by histamine. In further tests, the capsaicin-induced calcium influx can also be inhibited by SL. The immunofluorescence and molecular docking analysis indicated that SL acts as an inhibitor of transient receptor potential vanilloid-1 (TRPV1) activation to decrease calcium influx against stimuli. In summary, these results revealed that SL may inhibit histamine-induced itch by decreasing PLC/IP3R signaling pathway activation and modulating TRPV1 activity. This paper indicates that SL can be a useful treatment for histamine-dependent itch.

## Introduction

Itch (pruritus), a disagreeable sensation of the skin, is classified into two distinct types: histamine-dependent and histamine-independent. It is widely acknowledged that histamine is an endogenous molecule responsible for eliciting itch, acute inflammation, and other forms of rapid hypersensitivity reactions^1,2^. Mast cells that produce histamine are usually located in the upper layer of the dermis of healthy skin, where the host organism is confronted with extraneous bacteria and antigens. Upon stimulation, mast cells will secrete histamine as a response^3,4^. Unmyelinated C-fibres, which are not responsive to mechanical stimuli, can be used to treat histamine-induced pruritus or sensitivity. Additionally, although itch-sensing neurons possess a repertoire of receptors that allows for a degree of direct irritants detection, itch is often initiated by epidermal and immune cells, and passed to sensory afferents through endogenous itch mediators^5^. Researches over the past decade have shown that itchy sensation is not purely a neuronal phenomenon but a complex involvement of skin, immune, and neural processes to complete this enormous task^6,7^. Histamine also has the capacity to stimulate human keratinocytes through histamine receptors, resulting in the upregulation of inflammatory cytokine, anti-microbial peptide and matrix metalloproteinase (MMPs), which can lead to inflammatory skin diseases^8,9^. Studies have indicated that H1 and H4 histamine receptors are strongly associated with allergic conditions, skin inflammation and itching^8,10^. Besides, H1 and H4 receptor antagonists are able to lessen the scratching behavior of those suffering from atopic dermatitis (AD)^11,12^. Recent research has suggested that the use of next-generation anti-histamines that target both H1 and H4 receptors may be an effective treatment for skin irritation and itch^13,14^.

The transient receptor potential vanilloid-1 (TRPV1) channel, a type of non-specific cation channel, is activated when exposed to H^+^ ions, capsaicin, and heat, which has been suggested to participate in itchy sensations as well as the transmission process of pain^15^. Research has revealed that TRPV1 is expressed not only in the central nervous system and sensory neurons but also in human epidermal cells^15–17^. An immunofluorescence study reported in 2001 was used on primary keratinocytes from healthy individuals to detect the presence of TRPV1 for the first time. Subsequently, the primary keratinocytes is identified as the source of TRPV1^18^. Also, another research using in-vivo mice model only expressing epidermal TRPV1 gene has shown that the specific and selective activation of TRPV1 on keratinocytes is sufficient to induce pain and itch^17,19^. Studies conducted on cultured human epidermal keratinocytes (HaCaT) have demonstrated that the influx of calcium, mediated by TRPV1, can inhibite proliferation and induce apoptosis^13,20^. TRPV1 is also a key component in initiating physiological reactions following the activation of histamine receptors^15^. The activation of TRPV1 has been linked to the calcium-dependent production and release of signaling molecules such as PLC, IP3, diacylglycerol (DAG), and PKC^21,22^. It has been suggested that TRPV1 inhibitors can impede the increase of MMPs and interleukin 1β, 2, 4 caused by UV exposure, which are linked to inflammation^23^. Therefore, the activation of TRPV1 channel on keratinocytes is similar to that in dorsal root ganglion (DRG) and can be utilized as a target to gauge the intensity of itching.

Recently, there has been a surge in the search for biodegradable, natural and environmentally friendly ingredients for the treatment of pruritus. Previous studies have shown that Sophorolipid has the ability to alleviate inflammation, which is attributed to the suppression of the NF-κB pathway and the consequent reduction in the abundance of cyclooxygenase-2 and inducible nitric oxide synthase^24,25^. Our previous investigation has demonstrated that SL is effective in suppressing the calcium influx in RAW264.7 cells stimulated with LPS^25^. Therefore, it can be proposed that SL may have the capacity to inhibit histamine-induced calcium influx and be further applied as a treatment for skin deseases, such as pruritus. This study aims to determine whether SL has the potential to inhibit histamine-induced itch and affect the mechanics of histamine-induced intracellular calcium elevation in keratinocytes. We anticipate that these results will provide a new perspective for the application of SL in the medical and cosmetic field.

## Materials and methods

### Reagents

A non-pathogenic strain of Candida bombicola (ATCC 22214 from Shanghai Bioresource Collection Center, China) was used to produce SL through a fermentation process, as described in a prior study^25^. Histamine, loratadine, JNJ7777120 and capsaicin were obtained from Aladdin Biochemical Technology Co. Ltd. (Shanghai, China). HTMT and VUF8430 were purchased from Kuma Biochemical Technology Co. Ltd. (Shanghai, China). Fluo-4 AM, rabbit polyclonal TRPV1 antibody, Cy3-labeled goat anti-Rabbit IgG antibody and Hanks' Balanced Salt Solution (HBSS) were obtained from Beyotime Biotechnology Co. Ltd. (Shanghai, China).

### Cell culture

Human epidermal keratinocytes (HaCaT) were sourced from the BeNa Culture Collection (BNCC339817, Shanghai, China). The cells were cultivated in a modified version of Dulbecco's Eagle medium (DMEM, high glucose) (Gibco, Carlsbad, CA, USA) at 37 °C in a humidified atmosphere with 5% CO_2_ at 37 °C. The DMEM medium was supplemented with 10% (v/v) fetal bovine serum (FBS) (Gibco, Carlsbad, CA, USA). The cells were maintained by replacing the culture medium with a new complete medium every 2–4 days.

### Animals

Female mice of the C57BL/6 J inbred strain, aged 6–8 weeks, were procured from Shiyanjia Lab (Nanjing, China). Mice were housed in a temperature-controlled (22 ± 2 °C) and humidity-controlled (40–70%) animal room under a 12-h light/dark cycle, with free access to food and water. The study was performed in accordance with relevant guidelines and regulations of the Institutional Animal Care and Use Committee of the Jiangnan University. All experimental protocols were approved by the International Association for the Study of Pain. We followed the guidelines of ARRIVE (Animal Research: Reporting of In Vivo Experiments).

### Behavioral assays

The back area of mice ear (2 × 2 cm^2^) was depilated with electric hair clippers 24 h before starting the experiments. Mice were placed in a box (294 mm × 190 mm × 130 mm) for approximately 30 min for acclimatisation before each experiment. All drugs were injected intradermally with a 30G needle to the de-haired area behind the ear. Immediately after the injections, mice were recorded on video for 30 min and the number of scratch bouts counted in 5-min as a bin with an observer blinded to treatment. One scratch bout was defined as a lifting of the hind limb towards the injection site. The animals were divided into eight groups: four of them were used as control (blank, solvent, 0.5% SL, 1% SL) drug groups; the rest of them including histamine, and different concentrations of SL plus histamine were used to observe scratching behavior. In the inhibited experiments, SL (0.25%, 0.5%, 1% aqueous solution) was administered by intradermal injection 30 min before the subcutaneous injection of histamine (100 μM, soluble in deionized water). All drugs were injected in a volume of 100 μL.

### Histological analysis

Over the course of three days, a histamine injection was administered to the mice and after a 30-min interval, the animals were euthanized through intravenous injections of pentobarbital sodium. Subsequently, the skin sections with the scratching areas and adjacent regions were preserved in 4% paraformaldehyde for 24 h and then embedded in the paraffin wax. The hematoxylin and eosin (H&E) staining was used to visualize epidermal thickness and inflammatory cells infiltration. A light microscope was employed to view sections at a magnification of 400×.

For TRPV1 immunostaining, the skin segments of mice were first incubated with a primary rabbit polyclonal TRPV1 antibody (dilution factor: 1:100) overnight at 4–8 °C and then washed with PBS to remove the primary antibody. The sections were then incubated with the secondary Cy3-labeled goat anti-Rabbit IgG antibody for 1 h at room temperature. The nuclei were counterstained by 2-(4-Amidinophenyl)-6-indolecarbamidine dihydrochloride (DAPI) staining. All the sections were examined immediately and photographed with a laser confocal microscope (Leica TCS SP8, Vizsla, Germany).

### Spectrofluorometry

The influx of intracellular calcium concentration [Ca^2+^]i was quantified using the fluorescent probe Fluo-4 AM, a technique that has been reported in other study previously^25^. HaCaT cells were incubated in HBSS supplemented with Fluo-4 AM at a concentration of 5 μM and CaCl_2_ at 1.2 mM at 37 °C for a period of 30 min. Following Fluo-4 AM loading, the cells were washed three times with HBSS before being exposed to various samples for stimulation. The spectrofluorometer (Feyond-A300, Alll Sheng Co. Ltd., Hangzhou, China) was used to assess the fluorescence of Fluo-4-Ca^2+^ at excitation at 488 nm and emission in the range of 512–520 nm. Data was documented at intervals of 10 s.

### Fluorescence [Ca^2+^]i imaging

HaCaT cells were seeded in 6-well cell culture plates (10^5^ cells per well). After attaching, cells were incubated with HBSS containing 5 μM Fluo-4 AM and 1.2 mM CaCl_2_ at 37 °C for 30 min. After Fluo-4 AM loading, cells were washed 3 times with HBSS and were pretreated with different concentrations of SL (10, 15, and 25 μg/mL, containing 1.2 mM CaCl_2_) for 20 min before the end of the staining. Different stimuli were added to stimulate before utilizing the imaging system of the inverted fluorescence microscope (AE31E, Motic China Co. Ltd., Xiamen, China), then the 30th-second image after stimulation was captured and used to compare the fluorescence performance differences between the groups. According to the experimental conditions, a 10X magnification objective lens was used to shoot the image of the cells. For the analysis of fluorescent expressions, the self-updating package “Fiji”, a version of ImageJ containing several plugins that help in scientific image analysis, known from here on as "ImageJ".

### Real-time quantitative PCR

After the pretreatment with different concentrations of SL for 20 min, histamine was added to the SL + HIS group with its final concentration to be 100 µM, which was the same as the HIS group. Following 4 h of co-incubation in a 6-well plate, the Rneasy® RNA extraction kit (manufactured by Beyotime Inc., China) was utilized to isolated total RNAs. The cDNA was created through the use of a cDNA synthesis kit (manufactured by Beyotime Inc., China) on a T100 ThermoCycler (Bio-Rad, Hercules, CA, USA). The relative expression levels of mRNAs were calculated using the 2^‑△△Ct^ method, with GAPDH as the internal control for normalizing the relative expression. The sequence information of primers was as follows: GAPDH (F: 5′-GCGAGATCCCTCCAAAATCAA-3′; R: 5′-GTTCACACCCATGACGAACAT-3′); PLCγ1 (F: 5′-TCAAGTGTGCAGTCAAAGCC-3′; R: 5′-GCGCTCTTGATGAAGGTCAG-3′); IP3R (F: 5′-dAAGACGAGGAAGAGGTGTGG-3′; R: 5′-GTCCTCCTTCTGCCCTTCTT-3′); PKCα (F: 5’-GCTCCACACTAAATCCGCAG-3’; R: 5’-ACAGTCGTCGGTCTTTGTCT-3’).

### Immunocytochemistry

HaCaT Cells were cultured in 12-well cell culture plates (10^4^ cells per well). The cells were divided into five groups: Blank, Solvent (1% DMSO), CAP, SL, SL + CAP. After the pretreatment with different concentrations of SL for 20 min, capsaicin was added to the SL + CAP group with its final concentration to be 1 µM, which was the same as the CAP group. The duration of capsaicin stimulation is three minutes^26^. The SL group was only treated with different concentrations of SL for 20 min. Then, cells of all groups were washed once in PBS, fixed in fixative for 15 min, washed three times, added the immunostaining blocking solution and blocked at room temperature for 1 h. After first incubated with a primary rabbit polyclonal TRPV1 antibody (dilution factor: 1:100) overnight at 4–8 °C and washed with PBS to remove the primary antibody, the cells were then incubated with the secondary Cy3-labeled goat anti-Rabbit IgG antibody for 1 h at room temperature. The nuclei were counterstained by DAPI staining. At last, the slices were photographed by an inverted fluorescence microscope with a 10X magnification objective lens (AE31E, Motic China Co. Ltd., Xiamen, China).

### Molecular docking

For the purpose of docking analysis, the SL 3D structure (Compound ID: 117065237) was retrieved from PubChem (https://pubchem.ncbi.nlm.nih.gov/, accessed on 7 June 2022). The structure of TRPV1 protein (PDB ID: 3J5P) was sourced from Protein Data Bank (PDB, https://www.rcsb.org/, accessed on 15 February 2023). By using Discovery Studio software, we managed the process of eradicating water and ligand molecules from protein structures, as well as hydrogenation and charge manipulation. By utilizing Autodock Vina software with its default parameters, both the target protein and corresponding ligand were docked, thereby yielding all conceivable conformations. The free energy of the binding was employed to gauge the results.

### Statistical analysis

The data were expressed as mean ± standard error. The statistic analysis was performed using GraphPad. ANOVA was employed to evaluate the discrepancies between groups, and Tukey's post-hoc test was then employed for further analysis. A statistically significant difference was determined to be present when *p* < 0.05.

## Results

### SL alleviated histamine-induced scratching behavior in C57BL/6 J mice

**Figure 1:**
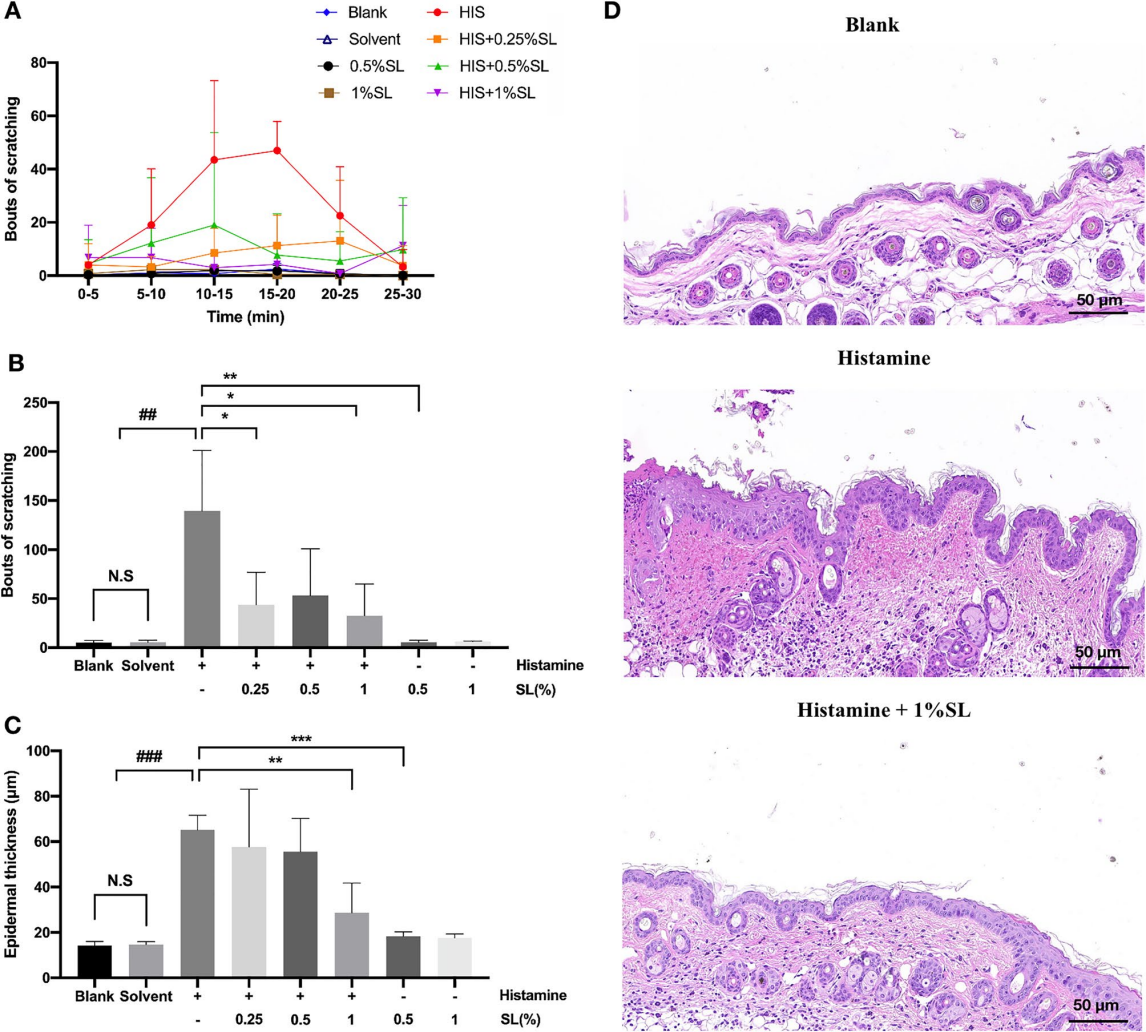
SL inhibited histamine-induced scratching in mice and affected the histopathology of skin lesions. Itch-related behavior was determined by bouts of scratching in 5-min as an interval during 30 min immediately following injection of pruritogens and vehicle. After three days of consecutive operations, the mice were euthanized. The animals were divided into eight groups: four of them were used as control (blank, solvent, 0.5% SL, 1% SL) drug groups; the rest of them including histamine, and different concentrations of SL plus histamine were used to observe scratching behavior. (**A**) A time-course graph for histamine-induced scratching between mice with or without pre-treatment of SL for 30 min. (**B**) Total bouts of histamine-induced scratching behavior with different doses of SL pre-treatment. (**C**) The epidermal thickness of the scratching site. (**D**) The representative images of H&E staining assay, scale bar = 50 μm. The results were represented as the mean ± standard error (n = 5). *p* < 0.05 was defined as statistically significant. (N.S., no significant; ###*p* < 0.001 vs. Blank and Solvent group; *, **, ***p < 0.05, 0.01, 0.001 vs. histamine treatment group).

Research has demonstrated that a specific dose of histamine can provoke a noticeable scratching response^13,27^. To investigate the antipruritic effect of SL, we administered 100 μL 100 μM histamine to C57BL/6 J mice to induce scratching behavior. As shown in Fig. 1A,B, it is apparent that between 10 and 25 min, the injection of histamine caused obvious scratching behavior and we found that this scratching behavior was inhibited by the pretreatment of SL. Even administration of the lowest dose of SL (0.25% aqueous solution) greatly reduced the total number of scratches caused by histamine. Our research indicates that SL has an inhibitory effect on scratching behavior caused by histamine.

The exposure to histamine is not only related with scratching behavior but also can result in damaged skin barrier and serious inflammation when skin pruritus worsens further. To further evaluate the subsequent impact of SL pretreatment on itchy skin induced by histamine, we conducted the H&E staining of the scratching site respectively. The histological analysis of the histamine group indicated intense inflammation, including epidermal hyperplasia, keratinization and inflammatory cells infiltration. If the skin barrier is damaged, the stratum corneum will be rapidly and compensatorily thicken^18^. Figure 1C shows that the epidermal thickness of the 1% SL-pretreated group significantly decreased from over 60 μm in the model group to less than 30 μm, which was comparable to the blank group. As shown in Fig. 1D, examination of H&E staining demonstrated that SL treatment reduced the epidermal thickness and improved the exfoliation of the stratum corneum compared with the histamine group. There results suggest SL can alleviate histamine-induced scratching behavior and subsequent skin inflammation in C57BL/6 J mice.

### Inhibitory effect of SL on histamine-induced [Ca^2+^]i influx

**Figure 2:**
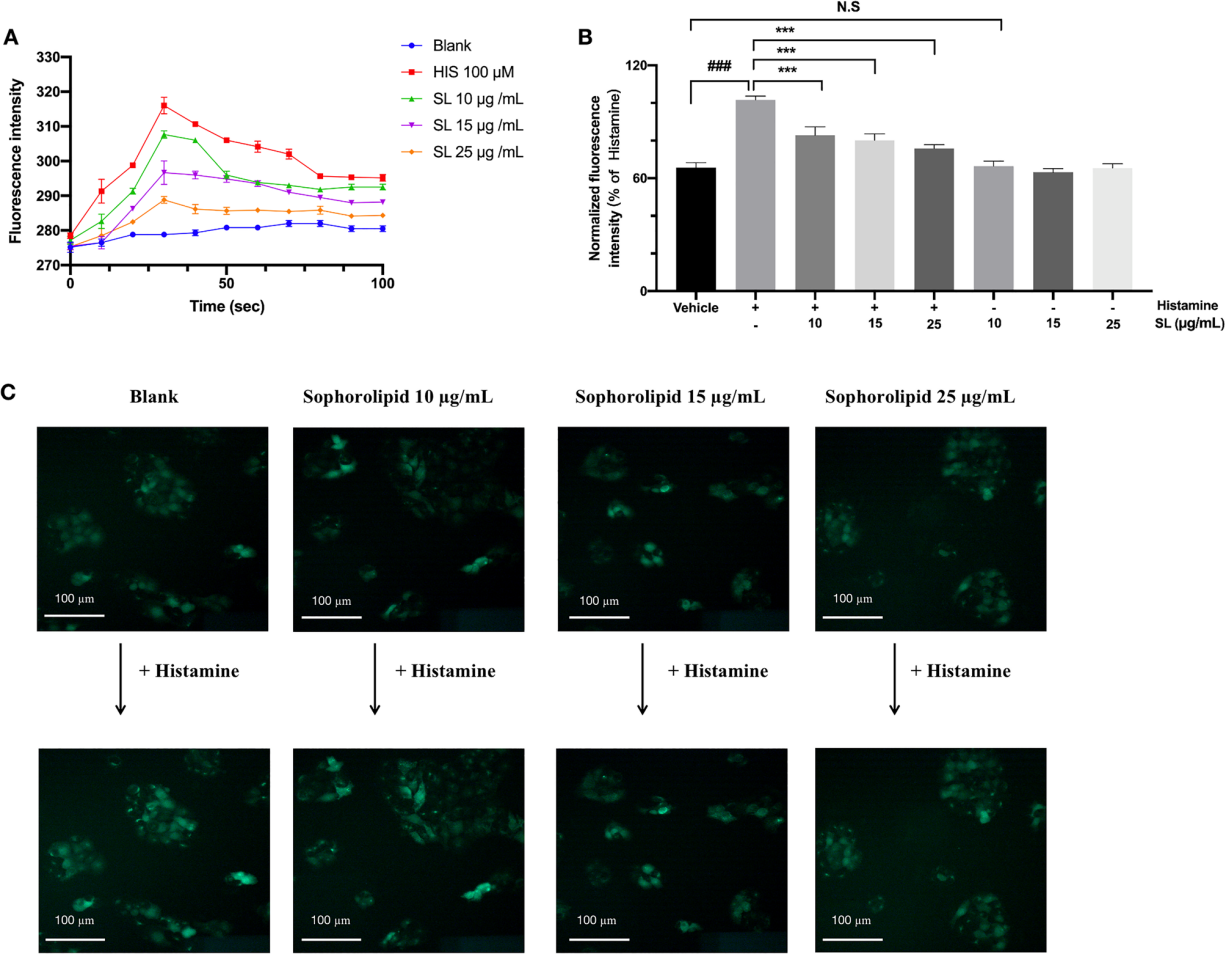
SL inhibited the [Ca^2+^]i elevation induced by histamine. (**A**) Fluorescence traces of cells’ response to 100 μM histamine (HIS) or 20 min pre-treatment of SL. (**B**) Effect of SL on the histamine-induced elevation of intracellular [Ca^2+^]i. SL inhibited [Ca^2+^]i influx in a dose-dependent pattern in response to 100 μM histamine treatment. (**C**) The representative images of SL suppressed the [Ca^2+^]i elevation using an inverted fluorescence microscope. The fluorescence intensity of Fluo-4 AM loaded HaCaT cells in response to histamine was conducted in the presence or absence of SL. The results were presented as the mean ± standard error (n = 3). *p* < 0.05 was defined as statistically significant (N.S., no significant; ###*p* < 0.001 vs. Vehicle group; ***p < 0.001 vs. histamine treatment group).

To investigate the mechanism of SL's antipruritic action, we developed an in-vitro cell model and employed [Ca^2+^]i influx as an indicator. As shown in Fig. 2, histamine (100 μM) was found to induce an obvious increase of [Ca^2+^]i concertration in HaCaT cells. The [Ca^2+^]i response consisted of an initial surge, a slow decrease, and a sustained phase. After pre-treatment with different concentrations of SL, the [Ca^2+^]i influx induced by histamine was significantly inhibited. Besides, this inhibitory effect of SL is dose-dependent at concentrations ranging from 10 to 25 μg/mL. The concentrations of SL and histamine, within the given range, were demonstrated to be non-cytotoxic for HaCaT cells, as described in the Supplementary Figs. S1 and S2. In order to see the inhibitory effect of SL more clearly, we applied an inverted fluorescence microscope to observe the change of calcium ions. As shown in Fig. 2B,C, we found that SL could significantly suppress the excessive [Ca^2+^]i influx caused by histamine and the intensity of the fluorescence weakened as the dose of SL increased. Also, from the histogram, applying different concentrations of SL alone does not stimulate intracellular calcium flow significantly compared to the Vehicle group (HBSS). This research confirmed that SL can inhibit calcium ion influx caused by histamine.

### SL affected the action of histamine receptors

**Figure 3:**
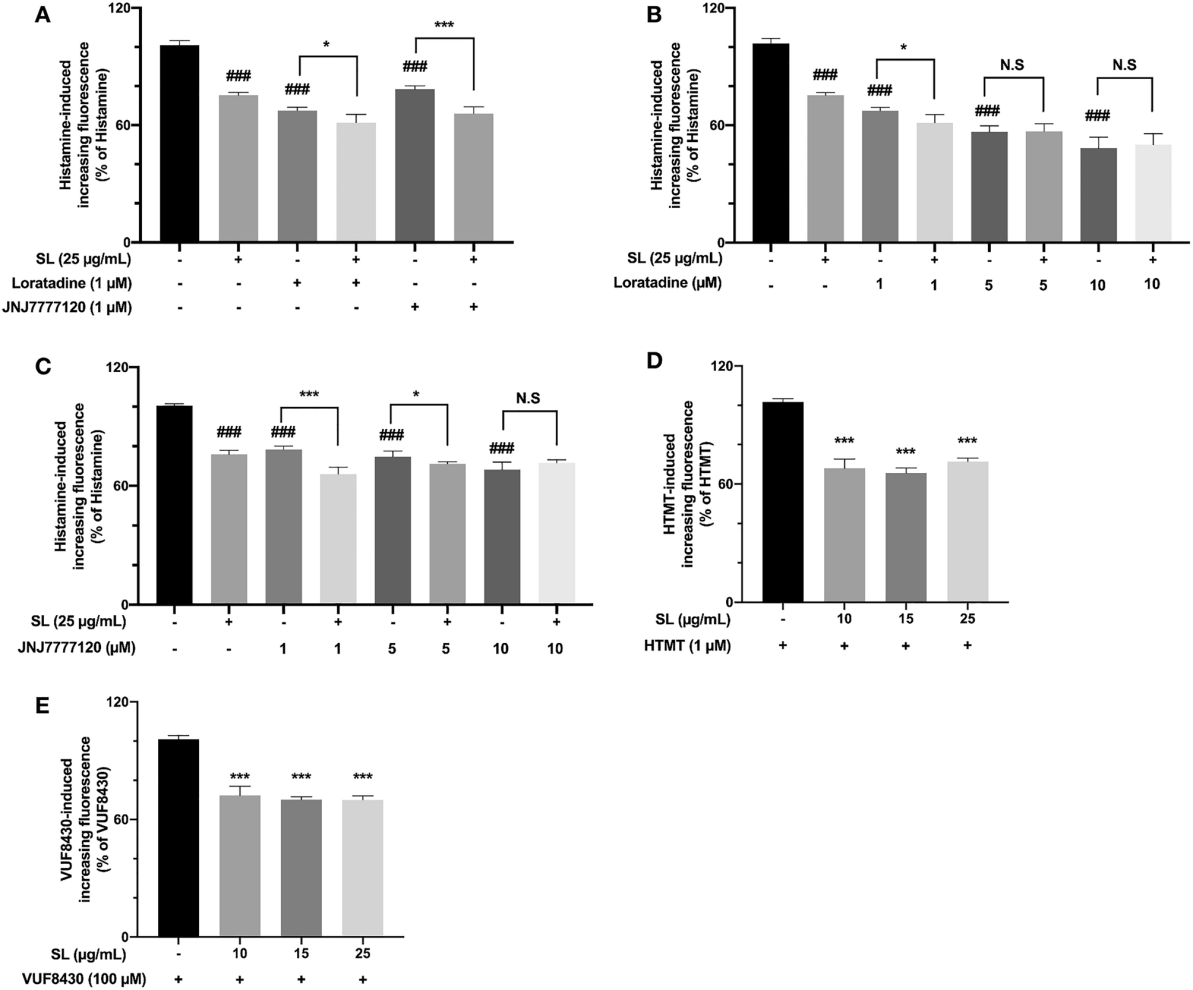
[Ca^2+^]i elevation induced by histamine or histamine receptor agonists (H1 agonist: HTMT; H4 agonist: VUF8430) was inhibited by SL or histamine receptor blockers (H1 blocker: Loratadine; H4 blocker: JNJ7777120). (**A**) The effect of SL on [Ca^2+^]i influx induced by histamine in the presence of Loratadine and JNJ7777120. (**B**) The inhibitory effect of SL in the presence of different concentrations of Loratadine. (**C**) The inhibitory effect of SL in the presence of different concentrations of JNJ7777120. (**D**) HTMT-induced [Ca^2+^]i elevation was inhibited by different concentrations of SL. (**E**) VUF8430-induced [Ca^2+^]i elevation was inhibited by different concentrations of SL. The results were presented as the mean ± standard error (n = 3). *p* < 0.05 was defined as statistically significant. (N.S., no significant; ###*p* < 0.001 vs. control group; *, ***, *p* < 0.05, 0.001 vs. agonist or blocker alone).

In normal human epidermal keratinocytes, studies have demonstrated that the use of histamine receptor blockers can suppress the histamine-induced calcium ion influx^13^. Therefore, we evaluated if SL can further reduce histamine-induced calcium ion influx in the presence of histamine receptor blockers. As shown in Fig. 3A, selective histamine receptor blockers could inhibit histamine-induced [Ca^2+^]i influx to varying levels. SL still inhibited histamine-induced [Ca^2+^]i influx when H1 or H4 receptor blocker was present. In addition, we investigated whether the inhibitory action of SL in the presence of H1 or H4 receptor blocker can be blocked in a dose related manner. As shown in Fig. 3B,C, the results indicated that while H1 or H4 antagonist can enhance its inhibitory effect with concentration increasing, the inhibitory action of SL can be weakened after co-incubation of 5 μM Loratadine or 10 μM JNJ7777120 respectively. These observations suggested that H1 and H4 receptor blockers both take part in the inhibitory effect of SL on histamine-caused [Ca^2+^]i elevation. Furthermore, we explored if SL can suppress the excessive intracellular calcium increased by specific histamine receptor agonists. As shown in Fig. 3D,E, the intracellular calcium levels, which were increased by H1 and H4 receptor agonist, were significantly lowered by different concentrations of SL respectively.

### SL inhibited histamine-induced PLC, IP3R, and PKC mRNA expression

**Figure 4:**
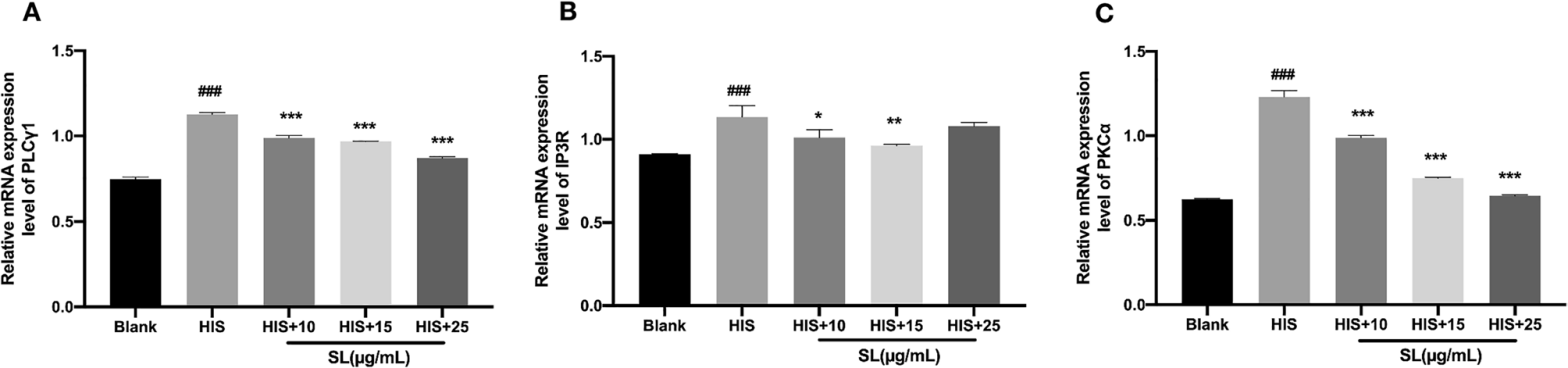
The effect of SL on the mRNA expressions of (**A**) PLCγ1, (**B**) IP3R and (**C**) PKCα in histamine-treated HaCaT cells. Cells were incubated with histamine for 4 h with or without a 20-min SL pre-treatment. Relative mRNA expressions were normalized to the corresponding GAPDH levels. The results were represented as the mean ± standard error (n = 5). *p* < 0.05 was defined as statistically significant. (###*p* < 0.001 vs. Blank group; *, **, ***p < 0.05, 0.01, 0.001 vs. histamine treatment group).

PLC has a crucial role in the signaling pathways of histamine-induced itch networks. Upon activation of H1/H4 receptors, PLC is stimulated, resulting in the generation of IP3 and DAG^28^ IP3 binds with the specific IP3 receptor (IP3R) and is implicated in the regulation of calcium ion flow. It is well known that IP3 and DAG are capable of triggering the activation of the pertinent PKC, a kind of signaling molecule which is also closely related to the transportation of calcium ions^21,22^. Both extracellular and intracellular sources of calcium ions are essential for initiating and maintaining the reactions. As shown in Fig. 4, mRNA expressions of PLCγ1, IP3R and PKCα were significantly increased by histamine, and SL inhibited the excessive mRNA expressions of PLCγ1 and PKCα induced by histamine in a concentration-dependent manner. As for IP3R, SL also apparently downgraded the mRNA expression at low concentrations.

### SL suppressed selective TRPV1 agonist-induced [Ca^2+^]i influx

**Figure 5:**
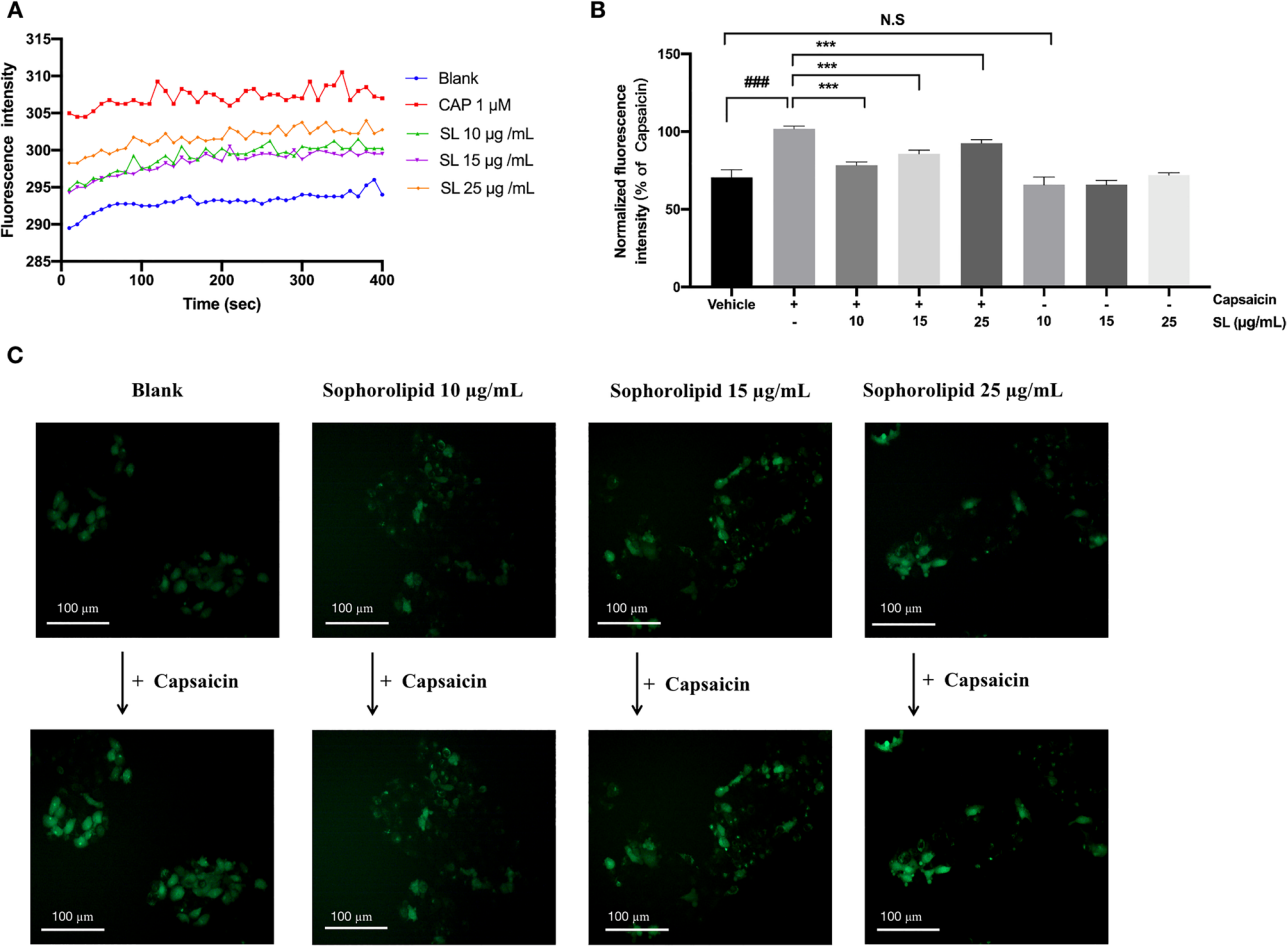
SL inhibited the [Ca^2+^]i elevation induced by capsaicin. (**A**) Fluorescence traces of cells’ response to 1 μM capsaicin (CAP) or 20 min pre-treatment of SL. (**B**) Effect of SL on the capsaicin-induced elevation of intracellular [Ca^2+^]i. (**C**) The representative images of SL suppressed the [Ca^2+^]i elevation using an inverted fluorescence microscope. The fluorescence intensity of Fluo-4 AM loaded HaCaT cells in response to capsaicin was conducted in the presence or absence of SL. The results were presented as the mean ± standard error (n = 3). *p* < 0.05 was defined as statistically significant. (N.S., no significant; ###*p* < 0.001 vs. Vehicle group; ***p < 0.001 vs. capsaicin treatment group).

To explore the impact of SL on the following downstream channels of histamine-induced fluctuation, we investigated the TRPV1 ion channel, a widely known pathway for calcium ion flow. Capsaicin was selected to be the activator of TRPV1 channel. As shown in Fig. 5A,B 1 µM capsaicin led to a significant increasing [Ca^2+^]i influx in HaCaT cells. After pre-treatment with various concentrations of SL, we could observe an obvious decrease in fluorescence intensity of [Ca^2+^]i; however, the inhibitory effect of SL was not dose-dependent. To better understand the inhibitory effect of SL, we found applying SL alone does not stimulate intracellular calcium flow significantly compared to the Vehicle group (1% DMSO). This implies that SL may potentially modulate the activity of the TRPV1 channel and then reduce the [Ca^2+^]i influx triggered by capsaicin.

### SL modulates TRPV1 activity

**Figure 6:**
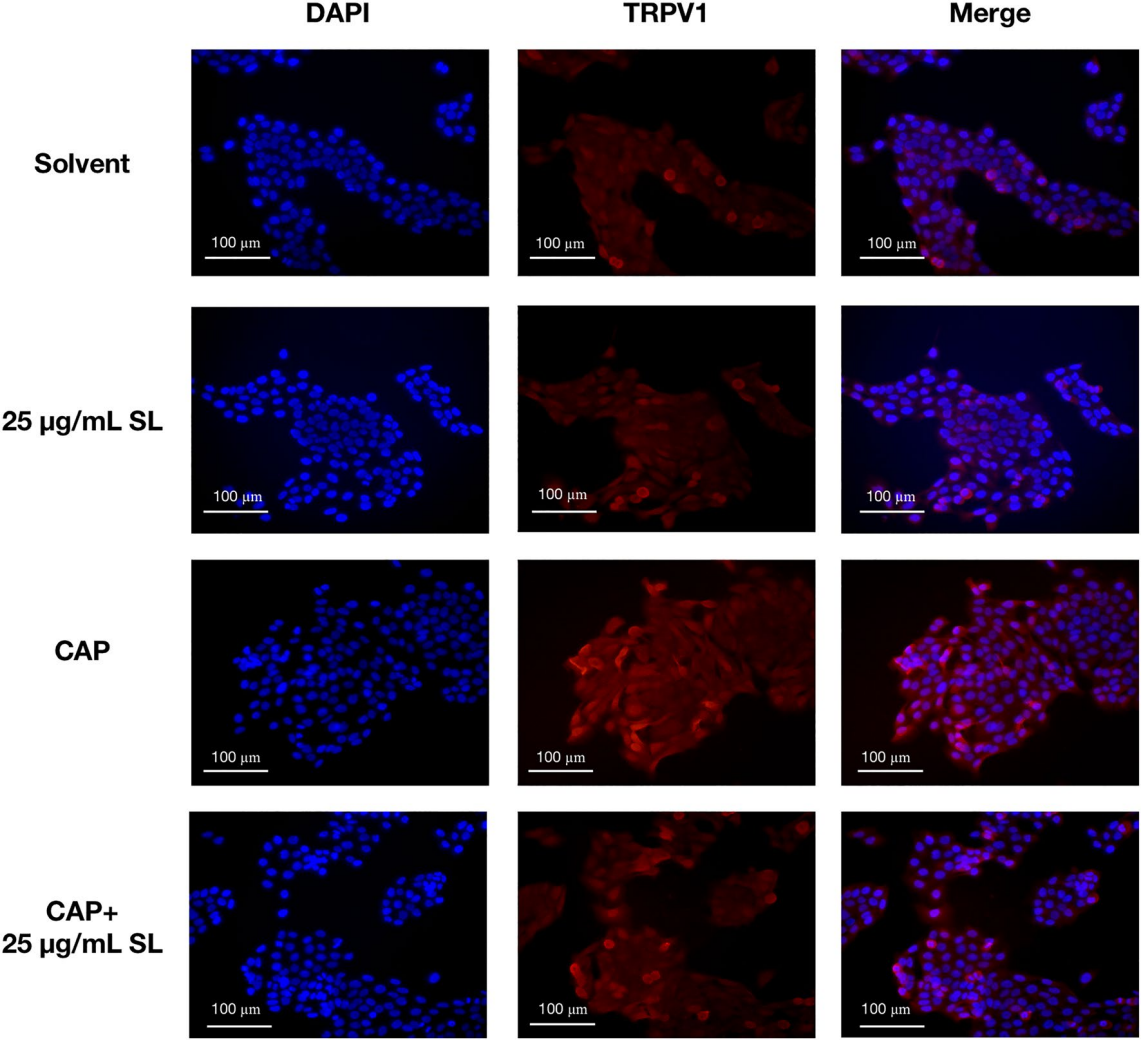
Immunocytochemistry detection in HaCaT cells indicated that SL inhibited the activiation of TRPV1 stimulated by capsaicin (1 μM). Localization of TRPV1 (red) and nuclei (blue) was visualized by an inverted fluorescence microscope after immunofluorescence staining. Results are representatives of three independent tests.

According to previous article^26,29^, TRPV1 can be detected in the cytoplasm and surrounding the nucleus of HaCaT by immunofluorescence. To further clarify the antagonistic effect of SL against TRPV1 activation, we used immunocytochemistry detection to mark the presense of TRPV1 protein in HaCaT cells. As shown in Fig. 6, TRPV1 were significantly activated when cells were treated with 1 μM capsaicin compared with the solvent group (1% DMSO). SL can inhibit capsaicin-induced upregulation of TRPV1 activation at a concentration of 25 μg/mL. Besides, treating cells with 25 μg/mL SL alone did not extra activate TRPV1 protein.

**Figure 7:**
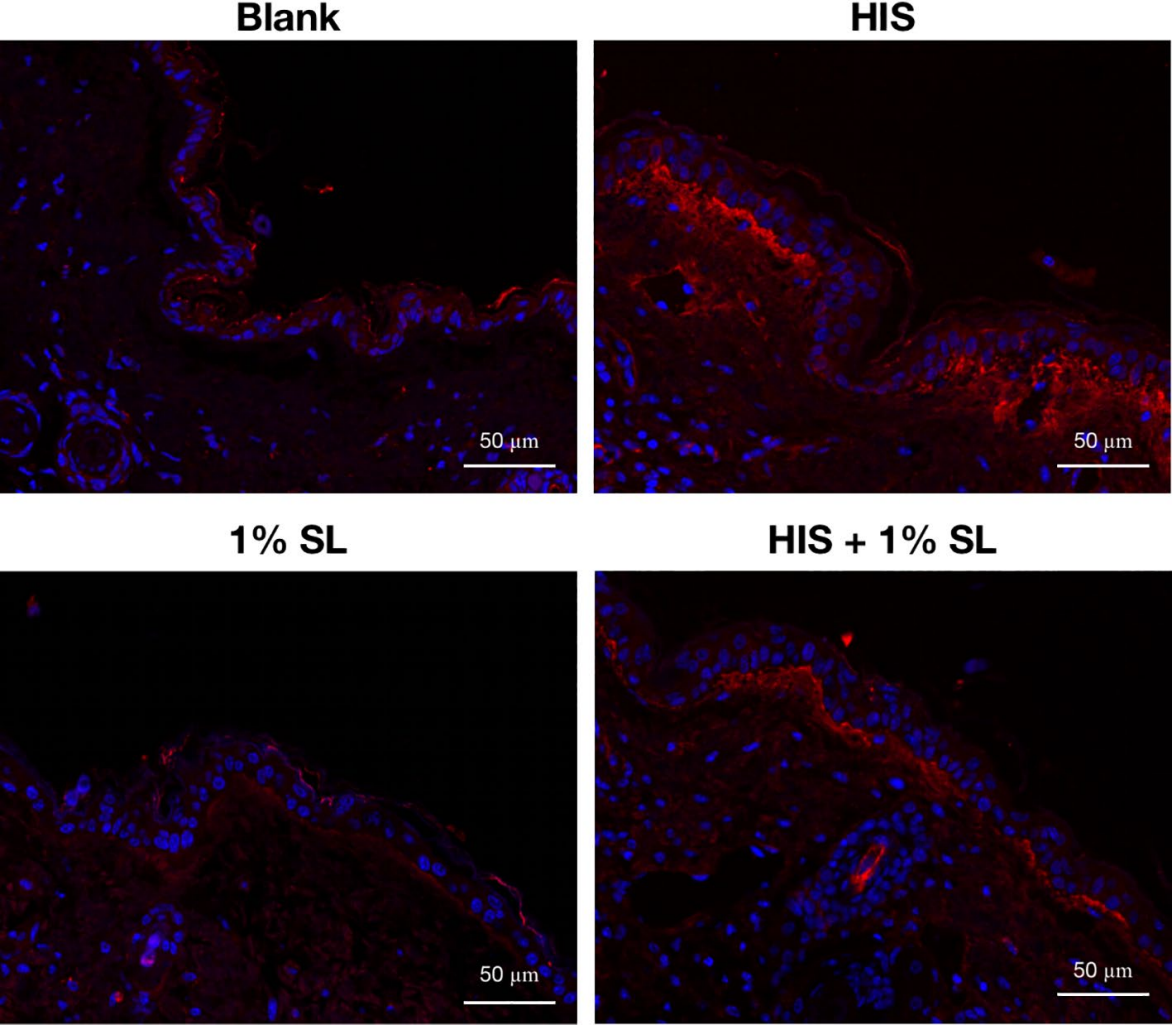
Immunohistochemistry detection in mice skin sections indicated that SL inhibited the activiation of TRPV1 stimulated by histamine (100 μM). Localization of TRPV1 (red) and nuclei (blue) was visualized by a laser confocal microscope after immunofluorescence staining. Results are representatives of three independent tests.

To examine SL’s action on histamine induced TRPV1 activation, we also conducted immunohistochemistry test of TRPV1 staining on mice skin epidermis after the mice were euthanized. As shown in Fig. 7, apart from damaged skin barrier and serious inflammation when skin pruritus worsened further, the exposure to histamine also resulted in strong TRPV1 activation compared with the blank group. However, 1% SL pretreatment inhibited this excessively activation of TRPV1 on mice epidermis.

The results of immunocytochemistry and immunohistochemistry indicated that SL can modulate TRPV1 activity against capsaicin and histamine.

**Figure 8:**
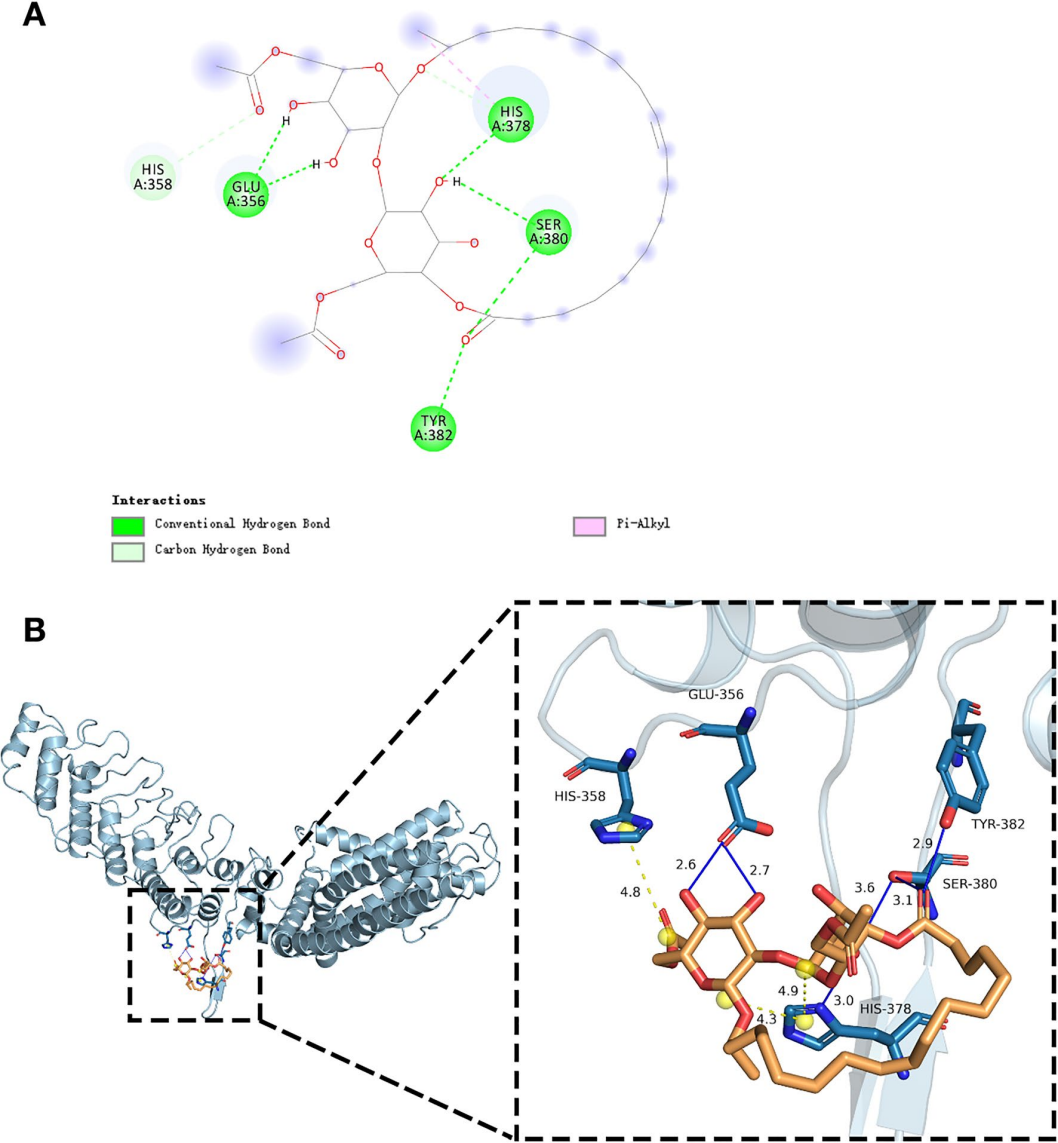
SL docked with TRPV1. (**A**) 2D structure of the docking interaction between SL and TRPV1. (**B**) 3D structure of predicted complex and the docking interaction of SL with TRPV1.

Finally, a molecular docking assay was used to determine whether SL has a direct interaction with TRPV1. The diacetylated lactonic SL ligand was docked with TRPV1 protein complex and the calculated binding free energy is -7.4 kcal/mol. As shown in Fig. 8A,B, the docking 2D and 3D stuctures indicate that the ligand-receptor connections are mostly contributed by conventional hydrongen bond with two glucose molecules of amino acid GLU 356, TYR 382, SER 380 and HIS 378. Besides, there exist a π-alkyl interaction of amino acid HIS 378 and a carbon hydrongen bond of amino acid HIS 358. The result is in accordance with the immunocytochemistry detection showing that SL may be able to bind with TRPV1 and inhibit the activation of TRPV1 induced by stimuli.

## Discussion

In recent years, there has been an increase in researches on the treatment of pruritus, an uncomfortable sensation on the skin. Suppression of itching is indispensable because it not only alleviates the unpleasant sensation but also help prevent further scratching, which is generally a sign of several common diseases, such as thyroid disease and atopic dermatitis. It is widely accepted that histamine is the main stimulus of allergic and itching reactions^1,8^. In medical clinics, anti-histamines are the most widely used medications to relieve itch^30^. A multitude of studies have demonstrated that natural plant extracts, such as osthole^27^, quercetin^13^ and red ginseng extract^14^ have the ability to suppress histamine-induced itch. This is the first investigation to suggest that SL, a kind of biosurfactant, can have an antipruritic action in C57BL/6 J mice against histamine, as well as alleviate further skin harm caused by scratching.

Histamine is identified when it binds to histamine receptors, initiating the downstream signaling pathway and subsequently activating a number of intracellular signaling molecules, such as the adenylate cyclase and PLC pathways^13,28^. The increase in intracellular calcium resulting from this phenomenon can lead to upregulated levels of of pro-inflammatory cytokines and may even induce apoptosis. It has been proved in previous studies that an overabundance of intracellular calcium can result in certain negative pharmacological effects associated with the receptor, the most common of which is pruritus^31,32^. The results of this article shown in Fig. 2 reported for the first time that SL has a dose-dependent capability to impede histamine-induced calcium influx.

Histamine action is widely recognized as being mediated by four distinct receptors, namely H1–H4^27,33^. Among these receptors, histamine H1 and H4 receptors are essential components in the transmit of histamine-associated itch signals between epidermal cells and peripheral sensory nerve endings^8,12,34^. Previous studies have demonstrated that neither histamine H1 nor H4 receptor blocker can block histamine-induced scratching behavior completely. Only when both histamine H1 and H4 receptor antagonists are associated with the transmission process of the itch signaling to the central nervous system can the histamine-induced scratching behavior be nearly suppressed^27,34^. Also, Itch caused by specific receptor activators cannot be inhibited by other receptor blockers. For example, thiopamine, a H3/H4 receptor antagonist, has been found to be ineffective in reducing scratching behavior caused by HTMT^35^. Extensive research has been conducted in recent years to elucidate the effects of H1 and H4 receptors on histamine-induced calcium influx both in vitro and in vivo models^10,11^. It has been demonstrated by various researchers that in human epidermal keratinocytes, histamine receptor blockers can also play an inhibitory role in histamine-induced calcium ion influx^13,22,32^. To ascertain if the inhibitory effect of SL is associated with histamine receptors, we conducted experiments to evaluate if SL can further inhibit histamine-induced calcium ion influx when histamine H1 or H4 receptor blockers are present. The observations in this study suggested that SL can affect the action of histamine H1 and H4 receptor. In addition, the increasing intracellular calcium induced by specific activation of H1 or H4 receptors can be reduced by different concentrations of SL. It is conceivable that SL may not act as a direct selective agent of H1 or H4 receptors, but instead, SL plays a partial role via the interaction with H1 and H4 receptors to inhibit the following signaling transduction.

After histamine binded with its specific receptors, the subsequent PLC, IP3, and DAG signaling elements are activated, resulting in the opening of corresponding calcium ion channels. Then this allows for the increase of calcium influx, which will lead to the generation of action potentials and neuro-sensitivity^21^. IP3 attaches to a particular IP3 receptor (IP3R) and is involved in the regulation of calcium ion movement. It has been reported that IP3 and DAG can stimulate the corresponding PKC, which is also linked with the conveyance of calcium ions^21,22^. We employed an RT-PCR analysis to examine the effect of SL on gene expressions that can be stimulated by histamine. Our research showed that histamine significantly elevated the mRNA levels of PLCγ1, IP3R, and PKCα and SL can reduce their expression to a certain degree.

TRPV1 has been well-acknowledged as a vital ion channel during the downstream signaling process of histamine stimulation^15,28^. Research evidence has indicated that TRPV1 is involved in the calcium response activated by histamine and the conduction of itch signals when the H1 or H4 receptor is stimulated^11,15^. We hypothesized that SL inhibits histamine-induced itching by modulating the activity of TRPV1, which was validated by demonstrating that SL was able to reduce the rise of [Ca^2+^]i caused by capsaicin (Fig. 5) and inhibit stimuli-induced activation of TRPV1 (Figs. 6 and 7). Molecular docking analysis also suggest that SL has a strong affinity for the TRPV1 protein complex and can act as an inhibitor by binding to the protein. These findings demonstrate that SL can suppress histamine-induced reactions by modulating TRPV1 activity.

Although our article found the antipruritic effect of SL with keratinocyte and mice models, there are several limitations in the current study. For example, the result of 0.25% SL in scratching behavior is not consistent with the skin tissue change, which needs additional verification to explain the phenomenon. In addition, the mechanism of itching is complex and we need to investigate more molecular pathways and establish different models such as sensory neurons to confirm the function of SL. Apart from histamine-dependent itch, there is also the presence of itch that is not related to histamine. Chloroquine, an anti-malaria drug, has proved to induce histamine-independent pruritus as a side effect^14^. Therefore, for a comprehensive understanding of SL's antipruritic effect, further examination on chloroquine-induced itch is required.

## Conclusions

This is the first research to indicate that SL, a kind of biosurfactant, is capable of alleviating histamine-induced itching by suppressing the activiation of the PLC/IP3R pathway and modulating TRPV1 ion channel activity in HaCaT cells, thus lowering the intracellular calcium level. This investigation suggests that SL may be a potential addition to medical and personal care products as it has an antipruritic effect and can help in relieving skin diseases.

## Supplementary Information

Supplementary Information.

## Data Availability

The data used to support the findings of this study are available upon request from the corresponding author.

## Data availability

The data used to support the findings of this study are available upon request from the corresponding author.
