# Supplementary material for: Sophorolipid inhibits histamine-induced itch by decreasing PLC/IP3R signaling pathway activation and modulating TRPV1 activity
Source: Sci Rep. 2023 May 17;13:7957. doi: 10.1038/s41598-023-35158-9 (PMC10192390; doi:10.1038/s41598-023-35158-9)
Supplement: Supplementary file 1 — Supplementary Information. [file 41598_2023_35158_MOESM1_ESM.pdf]

Sophorolipid inhibits histamine-induced itch by decreasing PLC/IP3R signaling pathway activation and modulating TRPV1 activity

Rui-Qi Xu[1], Ling Ma[2,*], Timson Chen[2], Wei-Wiong Zhang[2], Kuan Chang[1,*] Jing Wang[1,*]

[1] Key Laboratory of Synthetic and Biological Colloids, Ministry of Education, School of Chemical and Material Engineering, Jiangnan University, Wuxi 214122, China

[2] Adolph Innovation Laboratory, Guangzhou Degu Personal Care Products Co., Ltd., Guangzhou 510000, China

MTT Assay for Cell Viability:

After cells were treated with respective drugs for 24 hours, the culture-medium solution containing 0.5 mg/mL 3-(4,5-dimethylthiaxolone-2-yl)-2,5-diphenyl tetrazoliumbromide (MTT) (Sigma-Aldrich Co., Saint Louis, MO, USA) of 100 μL was added into each well, followed by another six hours of incubation at 37 ◦C. After that, the medium was carefully separated, and formazan crystals were solubilized by the addition of 100 μL of dimethyl sulfoxide (DMSO, Sinopharm Chemical Reagent Co., Beijing, China) into each well. An enzyme marker (Tecan Infinite 200Pro) was used to measure the absorbance at 490 nm. The cell viability was calculated according to the formula below:

Cell viability (%) = (OD_T/OD_B) × 100%, where OD_T and OD_B represent the average OD of the experimental group and the blank group, respectively.

Effects of Histamine on Cell Viability:

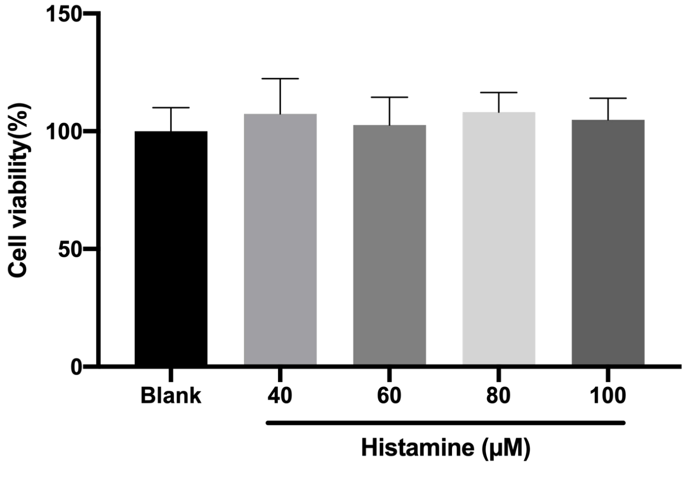

Figure S1. Effect of Histamine on the viability of HaCaT cells. After seeded into 96 well plates and incubated in an incubator for 24 hours, cells were treated with different concentrations of Histamine for another 24 h. The results are presented as the mean ± standard error of three independent tests in triplicate.

Effects of Sophorolipid on Cell Viability:

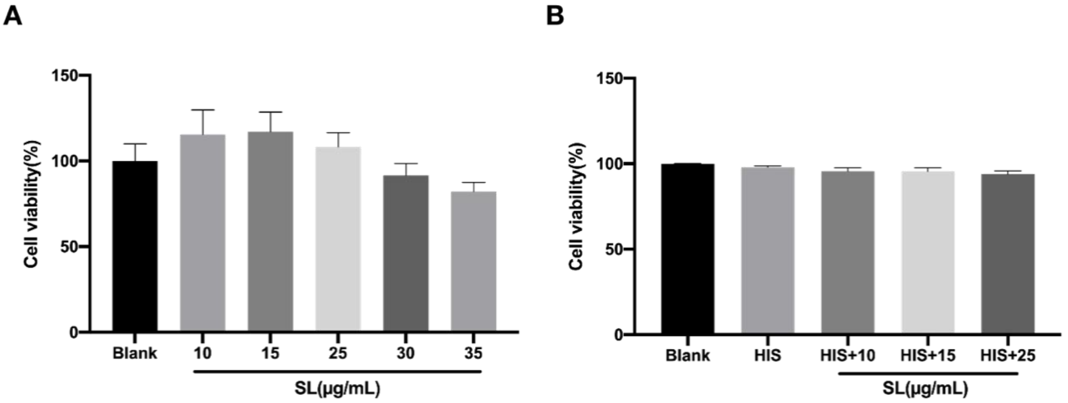

Figure S2. Effect of SL on the viability of HaCaT cells. Pretreatment with or without Histamine (100 μM) on cells for 1 h; then, cells were treated with different concentrations of SL for 24 h. (A) Cytotoxicity effect of SL on HaCaT cells. (B) Cytotoxicity effect of SL on Histamine-induced cell viability in HaCaT cells. The results are presented as the mean ± standard error of three independent tests in triplicate.

Fig. S1 and Fig S2 reveal that the cell viability was greater than 90% in the absence or presence of Histamine (100 μM), suggesting that SL with a concentration of up to 25 μg/mL had no cytotoxicity on HaCaT cells. Therefore, SL with a concentration of 10, 15, and 25 μg/mL was used in our subsequent experiments.
